# Trusting in the online ‘community’: An interview study exploring internet use in young people with chronic pain

**DOI:** 10.1177/20494637211061970

**Published:** 2021-12-27

**Authors:** Anna Hurley-Wallace, Sarah Kirby, Felicity Bishop

**Affiliations:** Psychology Department, 7423 University of Southampton, Southampton, UK

**Keywords:** Pain management, chronic pain, health psychology, internet use, qualitative research

## Abstract

**Background:**

Chronic pain in young people is prevalent in the UK. Young people are digital natives, yet there has not been any online intervention developed in a UK context to help them manage chronic pain. Key to understanding the context in which young people engage with online interventions is better understanding their internet use for chronic pain management. The overarching aim of this study was to explore young peoples’ experiences of searching for information about chronic pain using the internet. This included experiences of using search engines (e.g. Google), health information websites (e.g. the National Health Service [NHS] website) and social media (e.g. Facebook and Instagram).

**Methods:**

Semi-structured interviews were conducted with young people aged 16–24-years (*n* = 24), online, via Microsoft (MS) Teams. The study was advertised online and via patient partner charities. Interview data was analysed using reflexive thematic analysis.

**Results:**

Participants presented with a variety of chronic pain conditions, including joint hypermobility syndrome (*n* = 6), chronic headache and/or migraine (*n* = 4) and fibromyalgia (*n* = 3). Four themes were generated: ‘Trustworthy information, or experiences?’, ‘Diagnostic labels in a digital world’, ‘The online chronic pain community’ and ‘A mind and body approach to self-management’. Young people trust advice from others in their online community and having a diagnostic label help them find relevant pain management strategies and support networks online.

**Conclusions:**

This study is the first qualitative exploration of internet use in UK-based young people with chronic pain. Findings highlight the importance of considering internet use when developing new online interventions for young people with pain and that internet use, particularly social media use, is an important psychosocial consideration in pain management. Young people should be encouraged to verify practical pain management techniques found online with their doctor and be empowered in the safe use of appropriate psychology-based self-management resources.

Chronic pain in adolescence is a globally recognised problem.^1–4^ In the UK, 16–19% of adolescents experience multi-site chronic pain,^5^ which is associated with considerable functional disability.^6,7^ Interdisciplinary treatment^8^ reflects a biopsychosocial approach to chronic pain management and is recommended in practice for adolescents and young adults.^9–11^ Adolescence has recently been defined as up to 24-years-old, which reflects later social development.^12^ Indeed, during ‘late adolescence’, the impact of chronic pain is likely to result in delayed independence.^13^ Despite this, UK-based adolescents aged 16-years and over are considered independent with regards to healthcare.^14^

Online interventions for adolescents with chronic pain^15,16^ are increasingly used to support self-management and reduce strain on clinical services. In context of the COVID-19 pandemic, increasing access to evidence-based content for pain self-management, through technology, has become important.^17^ A recent trial of one such intervention, WebMAP,^18^ indicated that higher adolescent engagement with the intervention was associated with significant reductions in pain and disability.^19^

There has not been a multimodal, interdisciplinary online intervention developed for UK-based adolescents with chronic pain. Needs of adolescents in the UK may differ to adolescents in other western countries, based on their experiences of National Health Service (NHS) healthcare and experiences of chronic pain in different social contexts.^14,20^ Understanding context is important when developing complex healthcare interventions and qualitative research can provide insights into population-level factors that may impact intervention success.^21^ Experts agree that successfully designing online interventions demands a user-centred approach.^22^ The Person-Based Approach (PBA)^23^ provides an overview of how qualitative feedback from intervention stakeholders can be integrated into online interventions throughout development.^24^ The first stage (planning) focuses on conducting qualitative and mixed-methods research to understand the context in which users will engage with a novel intervention.

When developing online interventions for adolescents with chronic pain a key contextual consideration is how adolescents use the internet already in relation to pain management. This is important because adolescents aged 16–24-years are heavy internet users; 95% have a social media profile and 98% use the internet.^25^ Prior qualitative research has explored use of online resources for pain management in adolescents without chronic pain.^26^ Three themes were highlighted: drivers of internet use, barriers and anxiety around use. Anxieties included mistrust in the quality of online content, and some adolescents linked pain severity to their decision of whether to seek help in-person. Further, mixed-methods survey-based research has identified social media platforms, such as Instagram and YouTube, as important resources in chronic pain management for adolescents aged 16–18-years in the UK.^27^ However, pain-related internet use among older adolescents with chronic pain has not been explored qualitatively. Such research can provide insights into which resources are already being used and why. This research may reveal certain elements of pain management are not sought out or are already covered by existing resources.

The aim of this study was to explore the experiences of older adolescents (16–24-year-olds) with chronic pain when searching for information about chronic pain using the internet. This included experiences of searching the internet using search engines (e.g. Google), health information websites (e.g. the NHS website) and social media platforms (Facebook, YouTube and Instagram). Objectives were the following: (i) to explore young peoples’ experiences of chronic pain management strategies, including pain management techniques and advice provided by healthcare professionals, self-management strategies, and any internet resources that have helped facilitate this, (ii) to explore which resources young people believe have been the most helpful, and/or may have been potentially helpful for managing chronic pain, if available and (iii) to understand why certain resources are viewed as especially helpful for managing pain, or are noticeably popular, and why young people turn to these resources as opposed to, or as adjunctive to, in-person or online alternatives.

## Methods

### Study design

This study used semi-structured, individual interviews. Interviews were intended to be steered by the research question,^28^ and flexibly encourage talk about participants’ experiences.

Interviews were conducted online using Microsoft (MS) Teams during the COVID-19 pandemic in 2020. Participants also attended an initial online ‘screening’ interview. This familiarised interviewees with the software, addressed any concerns (e.g. data protection) and screened participants for eligibility.

This study was conducted from a critical realist epistemological standpoint. Critical realist epistemology suits the exploratory nature of the study objectives. Participant experiences were considered as approximations of reality, underpinned by existing social and psychological constructs.^29^ AHW’s role as an interviewer was anti-authoritative, viewing participants as experts on their own condition, only shifting to the ‘outsider’ role when clarifying health/medical terminology. Interviews were analysed using an inductive approach to reflexive thematic analysis.^30,31^

This article adheres to Qualitative Design Reporting Standards (JARS-Qual) (American Psychological Association).^32^

#### Researcher description

AHW is a PhD student specialising in adolescent chronic pain research (3-years’ experience) at the University of Southampton. AHW has an academic background in Health Psychology and has personal experience of chronic musculoskeletal pain diagnosed in young adulthood. AHW’s academic and personal background was explained briefly to all participants during screening. This may have enhanced rapport and encouraged storytelling during interviews. Personal experience may also have influenced data analysis, improving data immersion and leading to theme development through an empathetic lens.

The researcher had no prior relation to the study participants.

### Patient and Public Involvement

A Patient and Public Involvement (PPI) group was recruited, via the University Hospital Southampton and Applied Research Collaboration (Wessex), to assist throughout the study. The group consisted of three individuals aged 16, 22 and 27-years, who experienced, or had experienced, chronic pain throughout adolescence. They attended bi-monthly meetings on MS Teams to discuss aspects of the project and responded to WhatsApp queries. Contributions and selected illustrative changes included: reviewing the research protocol and study advertisements (which resulted in mentioning ‘social media’ in study advertisements), offering suggestions for the recruitment strategy and piloting the interview topic guide (which resulted in swapping the order of the sections Discussion and Conclusions).

### Recruitment

This study was approved by the University of Southampton Psychology Ethics Committee (reference: 56,803).

This study used convenience and purposive sampling. Specific ages and chronic pain types that were underrepresented in the sample were targeted after the guide target of 16 interviews (see ‘sample size’) had been met. Recruitment occurred from September 8, 2020 until December 9, 2020. The study was advertised online, via relevant charities and through a sixth-form college. Participants were offered £20 shopping e-vouchers for their time.

Online advertising included social media platforms Facebook, Instagram, Twitter, Reddit and LinkedIn (including Facebook and Instagram paid advertisements). Call for Participants (https://www.callforparticipants.com/) was used to co-ordinate social media advertising. Relevant charities were identified by AHW and the PPI group. The Hypermobility Syndromes Association (HMSA) (https://www.hypermobility.org/) was identified as a patient partner prior to the start of the study. Fibromyalgia Action UK (FMAUK) (https://www.fmauk.org/) were contacted following ethical approval. Both charities advertised the study via their respective websites and social media pages. AHW had personal contact with one UK-based sixth-form college. The study advert was circulated via email from the gatekeeper.

Potential participants that expressed interest were asked to provide their email address, were emailed the participant information sheet and were invited to a ‘screening’ interview. The participant information sheet explained how to access MS Teams.

### Participant selection

Participants with any type of self-reported chronic pain were eligible for inclusion in this study. Eligibility did not require a diagnosis of chronic pain by a healthcare professional, though clinically diagnosed conditions were noted.

Inclusion criteria: (i) 16–24-years-old,^12^ (ii) bodily pain lasting 3-months or longer total duration, (iii) chronic pain condition, including pain conditions listed on the chronic pain screening tool (Supplementary Material 1), or any other chronic pain condition diagnosed by a healthcare professional, as listed in the ICD-11,^33,34^ (iv) permanent residence in the UK (target group for exploring internet use in a UK context), (v) access to the internet for the online call and (vi) ability to communicate in fluent, spoken English.

This study initially aimed to interview approximately 16 individuals as a guide to achieve meaning saturation.^35^ However, the decision to stop recruiting was flexible, where AHW made an interpretative decision about when to stop coding and start generating themes.^36^ The main factor in this decision was high coding saturation observed between interviews with 16–17-year-olds verses 18 to 24-year-olds, following purposive sampling of younger participants, after high initial interest from older participants. However, as has been noted by others, reflexive thematic analysis cannot reach a fixed end point; new meanings are always possible.^37^

### Procedure

Interviews were conducted by AHW, online using MS Teams. All potential participants attended an initial screening interview, and eligible participants were invited to a research interview at a later date. Participants could choose to use video, or not, as suited their preferences.

Screening interviews lasted for approximately 10-minutes. This study used a verbal consent form, which was recorded at the beginning of the screening; the remainder of the screening was not recorded, to allow participants to become acquainted with the researcher and the online setting. Participants were screened using the demographic questions and chronic pain screening tool developed for this study (Supplementary Material 1, Supplementary Material 2).

For the research interview, participants were invited to attend an MS Teams meeting at a mutually agreed time. Participants were greeted, and audio/video consent was re-checked upon starting the recording. Interviews lasted for between 16 min and 72 min (M length = 35 min). The interview followed a semi-structured guide (Supplementary Material 3), and field notes were taken afterwards. At the end of the interview, the recording was stopped and participants were debriefed verbally and given opportunity to ask further questions. A written debriefing statement was emailed with the study reward after the interview.

#### Interview topic guide

An interview topic guide (Supplementary Material 3) was developed to ensure topics explored during interviews were consistent with the research objectives. Questions were asked in order by default; however, the order was used flexibly where participants naturally covered later topics. Topics that were initially skipped were returned to later on in the interview. Any other relevant topics that were brought into conversation by participants were explored as appropriate.

#### Recording and data transformation

Interview data collected in this study was initially reviewed by AHW using the video/audio recording and basic transcript, which is automatically generated in MS Teams.^38^ Video recordings were used to finalise field notes. Audio recordings were then extracted, and video recordings were destroyed. Audio-only recordings were pseudonymised, and then sent to an external provider for transcription. Names, locations and other identifying features were removed during professional transcription.

### Data analysis

Data collected in this study was analysed by AHW using an inductive approach to reflexive thematic analysis.^30,31^ Data was interpreted from a critical realist epistemological standpoint, hence existing social and psychological constructs underpinning participants’ accounts of experience were considered.^29^

Data analysis followed the six stages of thematic analysis.^30^ AHW read and re-read interview transcripts, and re-listened to the audio data, comparing this with field notes, and adding further notes as needed. Finalised transcripts were imported to NVivo 12.^39^ AHW then systematically generated codes, using an inductive, data-driven approach. Data was coded in meaning units and included in vivo codes. Existing codes were iterated throughout the coding process, and NVivo ‘memos’ were used to make notes about interesting features of the whole dataset.

Once coding was complete (coding manual provided in Supplementary Material 4), AHW searched for themes. Codes were collated into clusters of meaning to create candidate themes.^40^ Candidate themes were tested out in relation to the dataset and research objectives and then expanded upon using quotes to evidence claims. Themes were reviewed by FB and AHW to ensure overall fit to the coded dataset. Themes were iterated, a thematic map was created, and theme names and details were finalised. Lastly, the research team created this report (first draft by AHW).

## Results

Twenty-four UK-based participants (median age: 21-years, range: 16–24-years) were interviewed. A summary of participant characteristics is presented in Table 1. An additional four individuals were screened. One did not meet age criteria, one did not attend interview and two were screened and added to the wait-list during purposive sampling; no responses were received when later followed-up.

**Table 1.** Participant demographic and pain characteristics: descriptive summary.

	Number of participants	% Participants in each category
Age (years)		
16	1	4.2
17	2	8.3
18	2	8.3
19	2	8.3
20	1	4.2
21	5	20.8
22	1	4.2
23	3	13.5
24	7	29.2
Chronic pain type^a^		
Primary	13	54.2
Cancer-related	0	0
Post-traumatic	3	13.5
Neuropathic	0	0
Headache and orofacial	4	16.7
Visceral	1	4.2
Musculoskeletal	9	37.5
Pain duration		
⩾3-months	1	4.2
⩾6-months	2	8.3
⩾1-year	3	13.5
⩾3-years	6	25.0
⩾5-years	12	50.0
Gender		
Female	21	87.5
Male	1	4.2
Gender variant/non-conforming	2	8.3
Ethnicity		
White	21	87.5
Mixed/Multiple ethnic groups	0	0
Asian/Asian British	3	13.5
Black/African/Caribbean/Black British	0	0
Other ethnic group	0	0

^a^Pain types defined using ICD-11 criteria for chronic primary pain and secondary pain types.^33,34^ Five participants presented with two or more pain types.

Twenty out of 24 participants had a specific diagnosis, and two had idiopathic chronic pain (investigations ongoing). Two self-diagnosed participants were interviewed. Thirteen participants met criteria for primary chronic pain, and five participants presented with two or more pain types (Table 1). Specific diagnoses varied greatly, including joint hypermobility syndrome (*n* = 6), chronic headache and/or migraine (*n* = 4), fibromyalgia (*n* = 3), Ehlers–Danlos Syndromes (EDS) (*n* = 2), endometriosis (*n* = 1) and rheumatoid arthritis (*n* = 1).

### Thematic analysis

**Figure 1.**
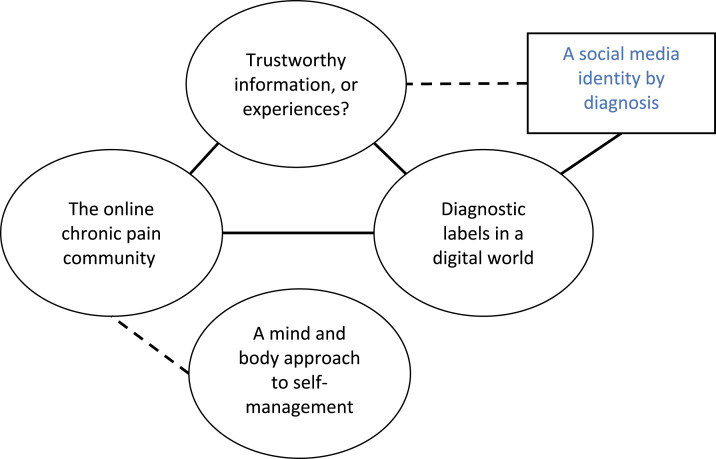
Thematic map. Themes are shown in ovals and subthemes are shown in rectangles. Direct connections between themes are shown using solid lines, and indirect connections are shown using dashed lines.

Four themes were generated: ‘Trustworthy information, or experiences?’, ‘Diagnostic labels in a digital world’, ‘The online chronic pain community’ and ‘A mind and body approach to self-management’. Within the diagnostic labels theme, the subtheme ‘A social media identity by diagnosis’ was also conceptualised. Figure 1 presents a thematic map.

#### Trustworthy information, or experiences?

The theme ‘Trustworthy information, or experiences?’ encompasses how adolescents and young adults use the internet to seek information about chronic pain and pain management. As might be expected within a group of young people who have grown up in a rapidly changing digital world, a variety of resources were talked about. Young people talked about using ‘trusted’ or ‘trustworthy’ health information resources, combined with information shared from an experiential viewpoint online, to shape their understanding of pain and pain management. In most cases, they turned to the internet first, before seeking advice in-person.

‘The first thing I did was Google, so the NHS resources, and from there I found the Hypermobility Syndromes Association, and then I found the Facebook support groups, which have been so, so useful’. – Cameron

How individuals reached the conclusion that resources were trustworthy was a central discussion. Young people described the NHS website as trustworthy because it is tied to the UK healthcare system, and they generally trust the healthcare system. Other information resources that were trusted included (i) charity-run websites, which were often linked via the NHS and (ii) health information websites that presented information from academic sources.

‘… the only ones I really use are like the EDS [Ehlers-Danlos Syndromes] website, NHS website, just the kind of main ones that I know I can trust, because I don’t want to be feeding myself false information’. – Laurie

‘I find the Fibromyalgia Action UK Facebook quite helpful sometimes, and they have really interesting articles about new ways of dealing with it’. – Cameron

Young people also sought information about chronic pain using videos posted by ‘professionals’ on YouTube. Here, there was discussion about ensuring the ‘professional’ was credible and ‘*actually qualified as what they’re claiming to be’,* (Eden)*.* Young people believed they could make informed choices about who to watch and listen to.

A key reason for diverting away from the NHS website, highlighted by several interviewees, was that it was lacking in detailed information on chronic pain and that the treatment options offered were ‘basic’ or ‘generic’. In reference to fibromyalgia, participants described the NHS website *‘missing a lot out’* (Alex) and providing nothing new. Many interviewees were inclined towards seeking pain management methods that they had not tried before, rather than re-trying any options listed by the NHS.

‘… in terms of treatment and stuff, I don’t think it’s updated very recently and it’s a very one road type approach to treatment, so it just like your typical pharmaceutical approach, your physio. I mean there are alternative approaches as well’. – Jamie

It appears young people’s motivation for turning first to the internet is because of the information available, which is continuously updated. Because they are keen to try anything that might help with pain, and the nature of pain is chronic, they are likely to continue circling back to the internet in the hope of finding new techniques to reduce pain.

Beyond typical health information-seeking, the internet was also used to search for experientially based advice on pain management, which was highly valued and generally trusted. Deciding who, and how much, to trust online was a complex process, which differed depending on whether the person being trusted was considered part of the online ‘community’ or not. This is discussed in greater depth in ‘The online chronic pain community’ theme (Figure 1). Trust in experience was, however, also present in relation to non-community platforms, such as blogs and forums. Use of these platforms to read about others tried and tested pain management strategies represents a different type of health information-seeking. Some pain management methods are chosen from the experience-base, where positive experiences were considered evidence of treatment success.

‘I would scroll through the pages on Google, looking for… not so much doctors or web page stuff, I wanted to see more what people had to say about it. It did take a while because I had to go through so many different pages just to find people that were relevant to what I had’. – Erin

In trusting others online, participants considered factors such as online identity, accuracy of experiential accounts, as well as advertising and ‘influencing’. Pain management strategies suggested online were sometimes verified for accuracy with a healthcare professional in-person:

‘Obviously, at the end of the day, these are all just strangers on the internet, so yeah, take everything they say with a pinch of salt but it’s nice to know that they’re out there and they have advice of what you could ask your own doctor’. – Harley

‘Trustworthy information, or experiences?’, is linked with the theme ‘Diagnostic labels in a digital world’ and indirectly linked to its subtheme ‘A social media identity by diagnosis’, where others’ advice seemed to be more readily trusted if they had an online identity that clearly featured chronic pain. Although, specific diagnostic labels were less important when trusting others within ‘The online chronic pain community’ (Instagram), compared to other user groups (e.g. Facebook groups and forums).

#### Diagnostic labels in a digital world

This theme emphasises a core message from young people with pain: relevant online information and support groups for chronic pain cannot be found without knowing what to look for.

A diagnosis of chronic pain was perceived as being crucial for ensuring accurate information about pain management strategies could be sourced online. This was typically emphasised in relation to information-seeking using internet searches (e.g. Google). Young people highlighted that once a diagnosis is made, this changes their internet search history, which becomes ‘tailored’ to the diagnosis.

‘I mean my own search history has changed since I was diagnosed, because I just never knew anything, like I hadn’t even heard of it’. – Alex

This focus on information-seeking around diagnosis links to a broader issue seen in young people with chronic pain, where experiences of diagnostic uncertainty are common. In synchrony with the rapidly changing digital world, this diagnostic search has become intertwined with internet use, where the diagnostic label becomes the search term.

Searching for a diagnostic label and ‘pre-diagnosis’ internet searching may fuel each other bi-directionally. Young people spoke of attempting to prompt a diagnosis from their doctor by ‘researching’ the suspected condition before appointments.

‘I found obviously the NHS page and just reading all the symptoms and it was just all adding up and “could it be something like this?” because it’s not like you can get a blood test for it and the doctors could just miss it. So, I booked an appointment, and I didn’t mention the fibromyalgia because I didn’t want to put something in the doctor’s head that might not be the case, but when I went to the doctor, I said about all my symptoms and the first thing he said was, “fibromyalgia”’. – Dylan

Participants compared searching based on a specific diagnosis to searching symptoms. The latter resulted in anxiety-provoking, worst-case scenarios appearing in the search results. Obtaining a diagnostic label ensures appropriate information resources can be found online and prevents escalation of symptom-based internet searching.

‘I think obviously it’s better once you get a diagnosis because… when you’re just searching like “pelvic pain”, “bad periods”, everything is coming up, it’s like, “You have cancer” and everything and you’re like, “Oh my god!” but now you actually know what it is, it’s a lot better because you can like search around your diagnosis’ – Frankie

#### Subtheme: A social media identity by diagnosis

Though the diagnostic label was often initially typed into internet searches to find accurate pain management information, the purpose of the search often shifted towards seeking experiential advice. Individuals were drawn towards online blogs and forums, as well as reading and listening to other young people’s experiences of chronic pain on social media platforms. ‘Diagnostic labels in a digital world’ has a different meaning in context of seeking support from others with lived experience; the diagnostic label becomes part of an online identity. For example, the label itself may be present in an Instagram or Twitter handle or may be used as a hashtag (#) to validate the pain condition.

‘I feel more valid by the fact that it’s a condition, beforehand you wouldn’t go on Instagram and just search like “pain” but if you follow people who have got like EDS [Ehlers-Danlos Syndrome] and people talk about that stuff’. – Toni

Engaging with social media, whether liking a video or sharing your own story, requires an online identity. Participants expressed the belief that having an online identity featuring chronic pain is essential for online group membership. The structure of social media may have contributed to such beliefs, where diagnostic labels are used to identify the group and its members. One young person spoke about membership of a ‘general’ group for pain, but legitimised the group by referring to those members who had a diagnostic label:

‘I’m part of, not one of the official groups but one that’s got quite a lot of members, people that have got hypermobility syndromes, EDS and people like that. And they have been so supportive when it comes to seeking help, and they’re often a lot more knowledgeable than regular people would be’. – Cameron

Young people also touched on the idea that *‘some people get very competitively ill’* (in vivo code – Toni), in that they may challenge others’ diagnoses online. Such challenges may increase the perceived need for a diagnostic label.

‘It can be quite draining just seeing people all the time being like, “Well I’ve got this” or, “I’ve got that” or downplaying people’s pain, being like, “No, you haven’t got that, yours isn’t as bad as mine”, that can be quite, I don’t want to say “toxic” but it’s not very good’. – Jamie

#### The online chronic pain community

‘The online chronic pain community’ highlights that young people believe the online resource that has been the most helpful to them for managing chronic pain is the Instagram ‘community’ of young people with chronic pain. Interviewees talked about using Instagram to seek information to help them understand chronic pain, and to seek practical pain management and empathetic support from others with lived experience. Though this ‘community’ is currently based on Instagram, it is likely to change as the popularity of different online platforms change over time. When accessing the online chronic pain community for pain management purposes, information-seeking and support-seeking became inseparable.

Being part of an online ‘community’ came across as distinctly different from being part of a user-group. Accessing forums was purposed as information-seeking, rather than seeking interpersonal interactions with other users. Whereas, in online communities, interpersonal exchanges of empathetic support and advice were central.

‘I just have learned a lot, especially about pain and the different kinds of pain as well and it’s really nice because I can relate with other people, so they’ll be like “I’ll have this and this” and then you sit there and think “oh my gosh yeah, that’s how I feel as well, that’s how my body’s feeling”, that’s really amazing, that’s probably been the best thing so far, this little Instagram community I’ve found’. – Dylan

Some individuals talked about using Instagram to branch out to YouTube and blogs for the purpose of reading or listening to another young person’s lived experience of chronic pain. Sometimes this was purposed as seeking advice; however, sometimes reading experiences of others from the ‘community’ was enmeshed with helping the young person feel less alone with pain.

‘Mainly social media and online, like the NHS website, because you do feel so alone, you want to find if there’s anyone dealing with the same thing and then if they’re having any successful methods of relieving it’. – Laurie

Thinking about the online communities outside of Instagram, there were suggestions that a safe space for young people with chronic pain to interact is needed. These suggestions were not specific to the pain condition, with emphasis that across conditions, the experience of being a young person with pain is shared.

‘… it would have been helpful to have a community of, like a safely accessible, because I was only so young, safe accessible sort of forums and sort of support group discussion type things for younger people experiencing it’. – Eden

Establishing an online community as ‘safe’ inherently links to the theme ‘Trustworthy information, or experiences?’ (Figure 1). The issue of online safety prompts questions about whether the person contributing to the community can be trusted; the accuracy of their online identity, and motivations for sharing information or advice. The above quote (Eden) emphasises that online safety is especially important in younger adolescence. Nonetheless, advice based on lived experience of chronic pain is embraced by young people in online communities.

Initial formation of online communities, and subsequent trust in online communities, may be fuelled by the underlying belief that the real-world perceives *‘young people shouldn’t have pain’* (Cameron), which was distinctly noticeable throughout interviews. Young people conveyed that their experiences of chronic pain are misunderstood by others in their social world and ‘dismissed’ by healthcare professionals.

‘Lots of family members have told me, “Oh, you’re so young, you shouldn’t have these issues, you’re so youthful, this is the prime of your life, why can’t you go and do this stuff?” and I’m like, “I physically can’t, my body won’t let me” this is a point of contention between me and my dad’. – Harley

‘I don’t really like to talk about it with people because they don’t really get it, and then I just feel really invalidated. I guess maybe a bit like alone from it? Yeah. Maybe a bit cut off from my friends’. – Charlie

These negative interpersonal experiences serve as an important context in understanding why many young people with chronic pain have turned to the online ‘community’; to seek validation and empathy from similar others. Supporting this interpretation, family members and friends who experience pain were favoured when seeking support in-person, because they are more understanding of the impact of chronic pain.

Feeling misunderstood by others corroborates findings from previous research on interpersonal relationships in young people with chronic pain. A qualitative synthesis of interpersonal relationships in adolescent chronic pain found discrepancies between adolescent’s and other’s perception of the impact of pain on daily life.^41^ Other research has found that adolescents with chronic pain are likely to interpret non-supportive social interactions with close friends as more distressing, and they tend to endorse, and expect, social support from friends.^42^ Young people in the current study additionally highlighted that they felt ‘dismissed’ by healthcare professionals, and this may further contribute to their search for validation and empathy through the online ‘community’.

Because part of the reason these communities have formed is to validate the pain experience, it is unlikely that advice based on lived experience of chronic pain will be rejected or questioned, because there is a great deal of shared empathy surrounding these experiences.

‘… the fact that all of us on the community have some kind of pain, we all kind of have that basic knowledge of what that’s like, and I don’t think there’s that many people with EDS and I feel like, to separate people with different things, even though we all have chronic pain, I think it would just make things complicated’. – Laurie

There is a clear link to diagnostic labelling here, where an online identity that includes chronic pain remains important to be embraced into the ‘community’ initially. Though, as per the quote from Laurie, and unlike ‘official’ groups (e.g. charity-led Facebook groups), less importance was placed on specific diagnoses in the ‘community’ context. This community, on Instagram or similar alternative platforms (depending on which is most popular at the time), is likely to continue growing as these young people act together to provide validation and support for each other.

Interestingly, some advice from members of the community drew from evidence-based information and analogies used in multimodal pain treatment, such as ‘spoon theory’ (used to explain fatigue) and use of Transcutaneous Electrical Nerve Stimulation (TENS).^43^

‘I learn a lot off [of] other people and I know a lot of people who I follow are in pain management, and they’ve got, they obviously see therapists and whatnot and so yeah, I guess that’s how they’ve learned, and they’ve put all their stuff on’. – Dylan

However, though intending to be helpful, some suggestions encouraged pain acceptance to the point of giving-up on functional improvements. For example, ‘balancing’ activities included accepting full-time employment was not realistic. This misconstrues how pacing would be used in clinical practice; pacing should be a stepped-progression towards improved functioning.^44^

### A mind and body approach to self-management

This theme encapsulates how young people use the internet to facilitate non-pharmacological pain self-management strategies. Specifically, young people talked about their use of the internet to improve any combination of their mood, sleep and physical activity levels as part of a general effort to manage chronic pain.

This theme can be indirectly connected to ‘The online chronic pain community’, as interviewees that were heavily engaged with social media talked about self-management in terms of what they had learnt from online communities.

‘… especially fibro and IBS, I’ve recently learned they’re so, so interlinked with one another and even how both of those are interlinked with how stressed you are, how much sleep you get, how active you are…’ – Dylan

Resources used to target mood and sleep that were mentioned frequently included mindfulness apps, Calm (https://www.calm.com/) and Headspace (https://www.headspace.com/), and YouTube. Young people identified that commercial apps can be expensive. Once the free components of commercial apps had been exhausted, YouTube was generally favoured for mindfulness, meditations and other relaxation resources.

Young people also used YouTube to participate in online yoga. YouTube yoga was talked about noticeably more than any other exercise technique. Aside from these classes being free and doubling as relaxation, an important context for the current study was the COVID-19 pandemic. It is likely that YouTube yoga became the most practical low-impact exercise choice for many people under ‘stay at home’ guidance.

‘I tried to do some yoga during lockdown and then walking my dog, if it’s not been too much, on days where the pain in my legs is more prevalent, my parents have taken my dog out for a walk instead of me, so I’ve tried to opt for a more easy form of yoga, just to kind of stretch and still do some exercise that isn’t too strenuous’. – Skye

One individual pointed out that exercising at home, using online resources, avoids feeling embarrassed about one’s exercise ability. This links to the belief discussed in ‘The online chronic pain community’, that *‘young people shouldn’t have pain’* (Cameron). In this case, the experience being misunderstood by others’ is the impact of chronic pain on exercise participation*.*

‘… everyone talks about things like do some strengthening activities like yoga or Pilates but when you’re in pain, you don’t feel like doing that so if there’s particular key aspects you could just do at home, or try to build up to without having the pressure of anyone else looking at you’. – Billie

## Discussion

This qualitative interview study explored internet and social media use by 24 older adolescents with chronic pain. Objectives included exploring information-seeking surrounding chronic pain, resources that have helped facilitate pain management, which resources have been the most helpful and why. Three closely interlinked themes were generated: ‘Trustworthy information, or experiences?’, ‘Diagnostic labels in a digital world’ and ‘The online chronic pain community’. A fourth theme ‘A mind and body approach to self-management’ was also identified and linked indirectly to the community theme. The subtheme ‘A social media identity by diagnosis’ was also generated under the diagnostic labels theme and linked to the trustworthy information theme. Interestingly, ‘A social media identity by diagnosis’ was not directly linked with the online community theme; shared experience of chronic pain was important above and beyond having an identity that featured a pain diagnosis (Figure 1).

The theme ‘Trustworthy information, or experiences?’ revealed that non-professional advice on pain management was sought out frequently. There was a strong sense of trust in others’ lived experience of chronic pain, which was expanded upon in the online community theme. Having an online identity that featured chronic pain was not necessarily important within the online community, though helped young people find the community in the first instance. An underlying purpose of the community seemed to be validation of each other’s experiences of chronic pain; hence, experientially based advice was readily trusted. Trusting advice given online is a complex issue, given that 16–24-year-olds are more likely than the average adult to agree that people should be able to post on social media anonymously, hiding their identity.^25^ This indicates young people are likely to take advice given by others online, regardless of legitimacy. These are ‘strangers on the internet’; therefore, advice should always be checked with a healthcare professional. Relaying this message to young people can be difficult, particularly as the tendency to believe online health misinformation is dependent on the individual and the context.^45^

Clinicians working in primary care may be best positioned to prompt young people to be cautious about taking advice found online. This may serve as a protective measure at the peak of internet searching; when pain is first labelled as chronic, and when patients are placed on waiting-lists for multimodal or psychological therapies. There are a variety of self-management resources available online that can help with pain through regulating mood, as discussed in ‘A mind and body approach to self-management’. Indeed, young people seem to acknowledge the link between pain and mood, and many already use mindfulness and meditation resources in their efforts to manage long-term pain. As there is currently no comprehensive pain management resource for young people on the wait-list for pain-specific therapies, clinicians should empower young people in the safe use of available psychology-based apps and websites to facilitate chronic pain self-management.

Within the theme ‘Diagnostic labels in a digital world’, young people portrayed a high focus on obtaining a diagnosis, and this was intertwined with internet use. Often the diagnostic label becomes the internet search term, handle or hashtag, and for some young people this becomes ‘A social media identity by diagnosis’. This extends findings from other qualitative research exploring diagnostic uncertainty in young people with chronic pain. Diagnostic uncertainty was experienced by nearly one third of adolescents in one study, and a diagnosis of idiopathic chronic pain is often not accepted.^46,47^ Adolescents often embark on a search for the ‘right’ diagnosis that continues for several years, despite physicians’ attempts to cease further diagnostic testing.^48^ A key question for future research is to what extent social media, which requires labels inherently in its structure, is fuelling young people’s search for a diagnosis with chronic pain.

The current study found that young people with pain turn to ‘The online chronic pain community’ partly because they feel misunderstood by others in their social world. They then use the ‘community’ to facilitate dynamic exchanges of information, advice and empathetic support online. Research has previously shown that young people with chronic pain may encounter difficulties in interpersonal relationships. As discussed under this theme, young people with chronic pain may struggle in their interpersonal relationships, and there are often discrepancies between adolescent’s and other’s perceptions of the impact of pain on daily life.^41^ The online ‘community’ serves the purpose of validating the chronic pain experience for young people, where they cannot find such validation or support in-person and similar others are present online. An interpretative case study, which investigated the appropriation of Instagram for adults with chronic illness^49^ similarly highlighted that emotional support exchanges and validation contribute to the appropriation of Instagram for illness management. Regardless of why young people turn to online communities for any form of advice or support with chronic pain, content moderation on social media remains a pressing issue.^45,50^ As aforementioned, it is important to remind young people to remain vigilant of health misinformation and not to endorse online advice solely based on shared empathy.

Internet use represents an important context for engagement with digital interventions; understanding context from users’ point of view can impact intervention success.^21–23^ Findings from the current study illuminate four points that should be considered by developers of online interventions for 16-–24-year-olds with chronic pain. First, young people are continuously seeking new pain management strategies; advertising an intervention as containing something novel that they have not yet tried may work as a ‘hook’ for initial engagement. Second, young people emphasise the importance of online communities to support everyday pain management, hence adding a community platform within interventions may improve engagement. Third, it is important that novel online interventions and resources are linked via a trusted source such as the NHS website. Fourth, young people are open to all types of chronic pain being addressed within the same platform; they identify that the overall experience of chronic pain is shared across diagnostic categories, and they may be more likely to embrace an intervention targeting their age group as opposed to specific diagnoses.

Limitations include that the sample did not represent young people with cancer-related or neuropathic pain, and disproportionality represented participants of female sex (only one male was interviewed). Nonetheless, a range of chronic pain conditions were represented. Two individuals who did not identify with a male or female gender were interviewed in this study; it is important the experiences of LGBTQA + young people are included in chronic pain research, especially where intervention is intended for use across genders.

## Conclusions

This study is the first qualitative exploration of internet use in UK-based young people with chronic pain; this is an important topic given the rapid expansion of digital healthcare over the past few years. This study highlights the importance of considering internet use in context of developing new online interventions for young people with chronic pain and the importance of considering young people’s internet use in clinical practice. Findings showed that young people tend to trust advice from others’ they consider to be part of their online community. Clinicians working with young people with chronic pain should advise them to check practical pain management strategies others suggest online with their doctor. There are a variety of psychology-based resources that young people should be empowered to use to help manage pain. Findings also revealed that having a diagnostic label helps young people find relevant support networks and appropriate pain self-management online.

## Supplemental Material

sj-pdf-1-bjp-10.1177_20494637211061970 – Supplemental Material for Trusting in the online ‘community’: An interview study exploring internet use in young people with chronic pain Click here for additional data file. Supplemental Material, sj-pdf-1-bjp-10.1177_20494637211061970 for Trusting in the online ‘community’: An interview study exploring internet use in young people with chronic pain by Anna Hurley-Wallace, Sarah Kirby and Felicity Bishop in British Journal of Pain

sj-pdf-2-bjp-10.1177_20494637211061970 – Supplemental Material for Trusting in the online ‘community’: An interview study exploring internet use in young people with chronic pain Click here for additional data file. Supplemental Material, sj-pdf-2-bjp-10.1177_20494637211061970 for Trusting in the online ‘community’: An interview study exploring internet use in young people with chronic pain by Anna Hurley-Wallace, Sarah Kirby and Felicity Bishop in British Journal of Pain

sj-pdf-3-bjp-10.1177_20494637211061970 – Supplemental Material for Trusting in the online ‘community’: An interview study exploring internet use in young people with chronic pain Click here for additional data file. Supplemental Material, sj-pdf-3-bjp-10.1177_20494637211061970 for Trusting in the online ‘community’: An interview study exploring internet use in young people with chronic pain by Anna Hurley-Wallace, Sarah Kirby and Felicity Bishop in British Journal of Pain

sj-pdf-4-bjp-10.1177_20494637211061970 – Supplemental Material for Trusting in the online ‘community’: An interview study exploring internet use in young people with chronic pain Click here for additional data file. Supplemental Material, sj-pdf-4-bjp-10.1177_20494637211061970 for Trusting in the online ‘community’: An interview study exploring internet use in young people with chronic pain by Anna Hurley-Wallace, Sarah Kirby and Felicity Bishop in British Journal of Pain
