# Supplementary material for: Trusting in the online ‘community’: An interview study exploring internet use in young people with chronic pain
Source: Br J Pain. 2021 Dec 27;16(3):341–53. doi: 10.1177/20494637211061970 (PMC9136991; doi:10.1177/20494637211061970)
Supplement: sj-pdf-1-bjp-10.1177_20494637211061970 – Supplemental Material for Trusting in the online ‘community’: An interview study exploring internet use in young people with chronic pain [file sj-pdf-1-bjp-10.1177_20494637211061970.pdf]

CHRONIC PAIN SCREENING TOOL

Non-diagnostic screening tool for chronic pain research

Participant ID: Click or tap here to enter text.

PAIN DURATION

☐ < 3-months
☐ ≥ 3-months
☐ ≥ 6-months
☐ ≥ 1-year
☐ ≥ 3-years
☐ ≥ 5-years

PAIN CONDITION*

☐ **Primary pain** including area-specific chronic pain of unknown aetiology (e.g. back pain, chronic widespread pain, fibromyalgia, IBS)

a. Primary pain is only appropriate where pain cannot be better explained by categories 2-7 and is associated with significant emotional distress or functional disability.

☐ **Cancer pain** - pain caused by cancer itself or by cancer treatments

☐ **Post-surgical or post-traumatic pain** - pain that persists beyond normal healing time following a surgical procedure or tissue injury

☐ **Neuropathic pain** - damage to the somatosensory nervous system.

a. Demonstration using imaging, biopsy, neurophysiological, or laboratory tests, in addition to negative or positive sensory signs, must be present for definitive identification as neuropathic.

☐ **Headache or orofacial pain** including primary and secondary headaches, and TMD/TMJ.

a. Pain must be present on at least 50% of days to be classified as chronic within this category.

☐ **Visceral pain** - pain originating from internal organs of the head and neck region and the thoracic, abdominal, and pelvic cavities

☐ **Musculoskeletal pain** - pain arising as part of a disease process that affects the bones, joints, muscles, or related soft tissues. This includes conditions of persistent inflammation, such as arthritis, as well as pain resulting from structural osteoarticular changes, such as EDS and joint hypermobility syndromes.

*Tick as many as apply

DIAGNOSIS

☐ GP
☐ Consultant (a medical doctor, e.g. paediatrician, rheumatologist)
☐ Nurse (clinical nurse specialist or advanced nurse practitioner)
☐ Physiotherapist/ physical therapist (e.g. occupational therapist)
☐ Somebody else Click or tap here to enter text.
☐ Self-diagnosed

DECISION

☐ Screening PASSED
☐ Screening FAILED
