# Supplementary material for: Trusting in the online ‘community’: An interview study exploring internet use in young people with chronic pain
Source: Br J Pain. 2021 Dec 27;16(3):341–53. doi: 10.1177/20494637211061970 (PMC9136991; doi:10.1177/20494637211061970)
Supplement: sj-pdf-2-bjp-10.1177_20494637211061970 – Supplemental Material for Trusting in the online ‘community’: An interview study exploring internet use in young people with chronic pain [file sj-pdf-2-bjp-10.1177_20494637211061970.pdf]

Demographic form

Participant ID: Click or tap here to enter text.

1. How old are you (in years)?

Click or tap here to enter text.

2. What sex were you assigned at birth, on your original birth certificate?

☐ Male

☐ Female

3. What is your gender?

☐ Male

☐ Female

☐ Transgender Male

☐ Transgender Female

☐ Gender variant/ non-conforming

☐ Other Click or tap here to enter text.

☐ Prefer not to say

4. What is your ethnicity?

☐ White

☐ Mixed/ Multiple ethnic groups

☐ Asian/ Asian British

☐ Black / African / Caribbean / Black British

☐ Other ethnic group

5. What is your postcode?

Click or tap here to enter text.
