# Supplementary material for: Trusting in the online ‘community’: An interview study exploring internet use in young people with chronic pain
Source: Br J Pain. 2021 Dec 27;16(3):341–53. doi: 10.1177/20494637211061970 (PMC9136991; doi:10.1177/20494637211061970)
Supplement: sj-pdf-3-bjp-10.1177_20494637211061970 – Supplemental Material for Trusting in the online ‘community’: An interview study exploring internet use in young people with chronic pain [file sj-pdf-3-bjp-10.1177_20494637211061970.pdf]

Supplementary Material 3. Semi-structured interview topic guide.

Question number	Questions and prompts
1	Can you tell me a bit about your experience of persistent/ chronic pain?<br>• What type of chronic pain have you experienced?<br>• How old were you when you first started getting pain?<br>• Can you tell me about what it was like when it first started?<br>• Can you tell me about what it has been like more recently?<br>• How does pain affect your school/ work/ university life?<br>• How does pain affect your physical abilities?<br>• How does pain affect your mood and emotions?<br>• How does pain affect your social life?
2	Can you tell me about any advice from doctors, nurses, psychologists, or physiotherapists that you have had about your pain?<br>• Can you tell me about advice you have had for your pain from any other alternative therapists or specialists?<br>• Can you tell me about your experience of treatments for pain?<br>• In the past, when you have been trying to find out how to deal with pain, who or where have you turned to first?<br>• Can you tell me about advice you have had about it from your friends and family?
3	What kinds of things do you do yourself to cope with persistent/ chronic pain/ your pain condition? (medication/ exercise/ relaxation/ mindfulness)<br>• What has that been like?<br>• Have you used any online resources to help you cope with pain by yourself? (websites/apps/media channels)<br>• Have you had any problems with the coping strategies you have used to help manage your pain?<br>• If so, how do you overcome these problems?
4	Can you tell me [more] about any internet resources you have used to find information about persistent/ chronic pain/ your pain condition?<br>• What are you hoping to find when you search the internet?

	• What are some of the things you have typed into a search to find out about pain?<br>• What things do you think about when you are looking for information about pain online?<br>• Can you tell me about any experiences you have had of using the NHS website to find out about pain?<br>• Have you used any other similar health advice websites (e.g., Healthline) to find out about pain, and what was this experience like?<br>• Can you tell me about any experiences you have had of looking for information about pain on social media (Facebook/ YouTube/ Instagram)?<br>• How has your use of internet resources and/or social media changed from when you were first diagnosed/ first started getting pain, compared to more recently?<br>• Which internet resources have you found the most helpful, and why?<br>• Which internet resources have you found were unhelpful, and why?
5	Thinking about online resources, is there anything else you think would be helpful, or would have been helpful in the past, for managing persistent/ chronic pain/ your pain condition?
