# Supplementary material for: Trusting in the online ‘community’: An interview study exploring internet use in young people with chronic pain
Source: Br J Pain. 2021 Dec 27;16(3):341–53. doi: 10.1177/20494637211061970 (PMC9136991; doi:10.1177/20494637211061970)
Supplement: sj-pdf-4-bjp-10.1177_20494637211061970 – Supplemental Material for Trusting in the online ‘community’: An interview study exploring internet use in young people with chronic pain [file sj-pdf-4-bjp-10.1177_20494637211061970.pdf]

Supplementary Material 4. Coding manual.

Name	Description	Files	References
1 good doctor can make all the difference	References to a particular doctor or healthcare professional being very important in making a difference to how pain is assessed and managed. Includes references to select GPs, physios, and other types of practitioners.	7	14
Academic sources - information-seeking	Information-seeking using academic sources such as Google Scholar or online journals. This could be information-seeking about the cause, symptoms, or treatment for a painful condition, which are often sought in parallel.	5	7
Accepting pain	Talking about being able to accept pain, in particular accepting the chronicity of the pain condition, and accepting some of the associated physical limitations. Some describe a sense of grief or loss associated with this, others have accepted it will always be there.	7	16
Accessing healthcare during COVID-19	References to not being able to access a variety of healthcare options during COVID-19, including face-to-face appointments, physical therapies, CAM.	9	19
Accessing NHS psychological therapies	Discussion of problems encountered when trying to access NHS psychological therapies/ talking therapies.	3	7
Accessing physiotherapy	Encountering problems with access or referral to physiotherapy for pain condition. Some discussion of being sent generic physiotherapy worksheets, which have not been helpful.	3	5
Advice from friends + family with pain	References to advice sought from or given by friends/family members who have experienced similar types of pain.	6	17

Name	Description	Files	References
Alternative health services	Use of healthcare services outside of primary/ secondary care, however not CAM or mental health. Examples: massage therapist, pharmacist, family planning, music therapy.	4	5
Anxiety-provoking content + worst-case scenarios	Anxiety-provoking content on the internet or an individual website. Includes extreme diagnoses popping up at the top of an internet search, and websites that relay worst-case scenarios for a condition. Examples of 'this could be cancer' or 'you are going to die of this illness' etc. Often associated with typing in symptoms rather than the pain condition itself.	10	21
Apps - distraction	Reference to specific apps that are used to facilitate distraction techniques.	2	4
Apps - exercise and stretching	Use of specific apps for exercising and stretching.	1	2
Apps - mental health - other	References to use of other mental health apps, coded separately to the meditation and mindfulness apps (Calm, Headspace).	2	7
Apps - organisation and reminders	References to use/ desire to use organisation and reminder apps to help with pain management.	2	6
Apps - relaxation, meditation, mindfulness	Use of apps such as Calm and Headspace for relaxation, meditation, and mindfulness. To help with pain or pain-related issues such as sleep.	10	29
Apps - symptom-tracking	Reference to using an unnamed pain symptom-tracking app.	1	1
Arranging pain management to reduce impact on exams	References to taking steps to ensure that pain management is up to date/ pain is minimised as much as possible in the run-up to academic	2	2

Name	Description	Files	References
	exams.		
Asking someone to help	References to having had to ask someone else for help, because the young person has been unable to complete a task themselves, due to pain.	4	4
Avoiding American websites because the health system is different to England	Reference to sticking to the NHS website as a primary source of information. Reasoned as the health systems are different in UK verses America and the treatment offered might be different, plus they trust NHS sources.	1	1
Avoiding medication where possible	References to avoiding taking medication where possible, or only taking medication when pain is severe. Usually related to worries over dependence/ tolerance or long-term impact of paracetamol/ NSAIDs.	12	17
Avoiding online groups for pain	Actively avoiding online groups for pain-related issues for a variety of reasons.	4	8
Avoiding social activities due to physical consequences	Talking about avoiding social activities, such as going out shopping, or travelling far, because of the anticipated physical consequences of increased pain. Advance planning of avoidance, as opposed to cancelling last minute.	6	7
Bad posture makes pain worse	Statement/ acknowledgement that bad posture can make pain worse.	3	3
Balancing your lifestyle at uni can be difficult	Talking about how balancing your lifestyle at university can be difficult and the impact that has on pain levels and pain management.	2	4
Becoming dependent on medication	Talking about being currently dependent or heavily reliant on medications/tablets.	3	5

Name	Description	Files	References
Before I had pain, I just didn't take people seriously	Reference specifically to not understanding others with pain or taking pain seriously, until they started experiencing pain themselves. Relates to 'lack of education and understanding of invisible illnesses'	2	3
Being aware of an injury during recovery	Reference to being 'aware' of a specific injury whilst recovering, however still keeping active.	1	2
Blogs - information-seeking	Information-seeking using online blogs. This could be information-seeking about the cause, symptoms, or treatment for a painful condition. Information sought is from the personal, experiential perspective of the writer.	2	3
Blogs - reading about others' experiences	Reading about other's personal experiences with pain via personal online blogs. These are not interactive.	1	2
Books + leaflets - information-seeking	Information-seeking using traditional paper resources such as books and leaflets. This could be information-seeking about the cause, symptoms, or treatment for a painful condition, which are often sought in parallel.	2	2
Catching COVID-19 would affect me more than others	Reference to worry that catching COVID-19 would affect them more than other healthy young people, hence avoiding going out completely.	1	1
Charity websites - information-seeking	Information-seeking on charity websites. This could be information-seeking about the cause, symptoms, or treatment for a painful condition, which are often sought in parallel. Some charity websites are linked via the NHS website.	10	16
Charity websites - unhelpful	Reference to contacting a charity website for support and the response being unhelpful.	1	1

Name	Description	Files	References
Choosing strategies that feel familiar	Choosing pain management strategies that feel familiar e.g. I used to dance, so I use the dance stretching app.	1	1
Complementary and alternative medicine	References to complementary and alternative medicine, either that they have tried or that they wish to try/ have been recommended.	11	18
Coping with pain	References to trying to cope/ manage or deal with pain, comparably to masking it with pain medications or trying to alleviate pain completely.	18	33
Creating a safe space for young people with pain	Expressions of a need for a safe space for young people with chronic pain to interact online.	3	8
Current study prompted online exploration	Expressions that the current study has prompted them to explore more online communities and website/ app options for dealing with pain.	3	5
Describing an injury	Describing a specific injury and how it occurred.	6	12
Describing pain location	Description of the location of pain on the body.	14	18
Describing pain sensation	Describing the feeling of pain or the pain sensation e.g., burning, aching, sharp etc.	13	30
Desire to improve self-management	Expressions of a desire/want to improve their self-management of pain in general.	6	10
Diagnosis is the key to finding accurate information	References to a diagnosis of pain or a painful condition being key/ crucial to finding accurate information and relevant support groups online.	11	23
Different medications for migraine vs joint pain	Reference to using different medication strategies for chronic migraine verses joint pain.	1	1

Name	Description	Files	References
	Comparing the different ways of using the 2 different medicines.		
Distraction techniques	Use of distraction techniques/tasks to draw attention away from the pain.	5	7
Doctors can misinterpret things	Talking about misinterpretation of symptoms or wrong diagnoses given by doctors. Some overlap with 'taking online information to the GP', however more emphasis on that symptom have previously been misinterpreted.	4	7
Doesn't use online resources for pain	Statement that the individual does not use online resources - websites/apps - for pain management.	2	4
Dropping out or delaying school or uni	References to dropping out of school or university or delaying/deferring by a year or longer due to pain-related issues. Some overlap with 'struggling to keep up with school/uni' - these are the more extreme cases.	4	4
Dyslexia and hand pain affect my ability to do exams	Specific reference to combination of dyslexia and hand pain impacting ability to succeed in school/ university exams.	1	1
Early prevention resources for chronic pain	Expression of a need for early prevention resources for chronic pain e.g., apps/ websites.	1	4
EDS website - provision of resources to help others understand	Reference to the Ehlers-Danlos website providing educational resources that can be used to help other people understand the condition.	1	2
EF - feeling alone	Emotional functioning - references to feeling alone or lonely. Relates to the code 'people around me don't understand' (feeling matches with the thought).	8	12

Name	Description	Files	References
EF - feeling anxious or stressed	Emotional functioning - references to feeling anxious, stressed, or worried. Some cases state this in reference to mental health comorbidities, however some express this as a feeling on its own.	7	9
EF - feeling low, down or upset	Emotional functioning - references to feeling low, down, upset, or similar.	13	15
EF - low motivation due to pain	Specific reference to having low motivation to get up and do anything when the pain is high.	1	1
'Even though I was in pain, I could have done more'	In vivo code. Talks about wishing they had put more effort into managing pain when they were younger/ at the start.	1	3
Facebook - information-seeking	Information-seeking using Facebook. Social media information-seeking often involves seeking experiential advice from others about symptoms and/or treatments for a painful condition.	5	11
Facebook - mainly for older adults	Reference to Facebook/ Facebook groups being for middle aged/ older adults, which has little appeal to young people.	1	1
Facebook - reading about others' experiences	Reading about other's personal experiences with pain via Facebook (pages or groups) or reading conversations/ interactions between other users about their pain experiences. Emphasis on reading/ liking/ viewing rather than sharing one's own experience.	3	7
Facebook - support groups	References to empathetic and interactive support groups on Facebook. Reading about other's experiences, rather than actively being part of a support group is coded separately.	8	24

Name	Description	Files	References
False advertising of 'cure all' tablets	Reference to false online advertising of 'cure all' tablets.	1	2
Feeling dismissed by a consultant	Talking about a feeling of dismissal or not being taken seriously by a consultant doctor at pain-related appointments.	3	7
Feeling dismissed by a physiotherapist	Talking about a feeling of dismissal or not being taken seriously by a physiotherapist(s) at pain-related appointments.	1	3
Feeling dismissed by GPs	Talking about a feeling of dismissal or not being taken seriously by GPs at pain-related appointments.	16	34
Fibro pain doesn't damage anything in the body	Statement that they know pain from fibromyalgia is not causing any physical damage in the body, and that exercise and activity is okay to participate in.	1	1
FMAUK website - outdated	Reference to Fibromyalgia UK website needing updating, more information, better layout.	1	1
Forums - information-seeking	Information-seeking using online forums or message boards. Information-seeking on forums often involves seeking experiential advice from others about symptoms and/or treatments for a painful condition (similarly to social media).	1	4
Forums - reading about other peoples' experiences	Reading about other's personal experiences with pain using online forums or reading conversations/ interactions between other users about their pain experiences. Emphasis on reading/ liking/ viewing rather than sharing one's own experience.	4	11
Giving up a sport due to pain	References to giving up a specific sport or needing to switch to an alternative, lower-impact	8	14

Name	Description	Files	References
	sport because of pain/ pain-related issues.		
Google or search engines - information-seeking	Information-seeking using Google or an alternative search engine. This could be information-seeking about the cause, symptoms, or treatment for a painful condition, which are often sought in parallel.	21	52
Google or search engines - parents information-seeking	Parental information-seeking using Google or an alternative, in replacement of the adolescent information-seeking themselves, regarding the cause, symptoms, or treatment for a painful condition.	1	1
Google or search engines - reading about others' experiences	Reading about other's personal experiences with pain via a variety of websites listed on an initial Google search or reading conversations/ interactions between other users about their pain experiences. Emphasis on reading/ liking/ viewing rather than sharing one's own experience.	1	1
GOSH physiotherapy intensive course	Discussion about specific experience of the Great Ormond Street Hospital intensive physiotherapy course (paediatric).	1	2
GP treating the immediate problem over chronic pain	Reference to GP treating acute diagnoses/ problems over addressing the chronic pain itself.	1	1
HCPs don't understand my condition	Reference to healthcare professionals not understanding EDS and proceeding to refer the individual around in a circle because they do not know how to treat.	1	4
High internet use at the start of the condition	References describing high/ much more internet use at the beginning/ onset of the pain condition compared to now. Some reference to that the information being sought about the pain	4	4

Name	Description	Files	References
	condition has been found, hence there is no need to search further/ as often.		
High pain during interview	Expressions of experiencing high severity of pain during the interview.	3	5
Hoping for a cure	References (often in vivo) to hoping to find a cure when searching online or hoping to find someone that has had the same condition and been cured. Most are aware that this is an unrealistic expectation and describe it as an underlying hope. 1 or 2 references, however, state they still believe there will be a cure for their pain.	7	16
I can join in by speaking to my friends online	Talking about feeling included in social interaction because they are able to speak to their friend online, WhatsApp, etc., even if they aren't able to meet in person.	1	1
'I can't concentrate as well as I could'	In vivo code. References to concentration levels being diminished by pain.	8	11
I can't do everything a normal teenager can do	States that pain is holding them back from being a normal teenager, and that they should be able to do everything.	1	1
'I can't go out as much as a normal 21-year-old would'	In vivo code. References to not being able to go out as much as other young people who do not experience chronic pain.	14	18
'I do worry about being consumed by constantly looking'	In vivo code. Expressing worries that online searching of symptoms, diagnoses etc. will become a mentally consuming, unhealthy habit.	2	2
I find the same online resources now as I did before	Reference to the online resources coming up when searching for information about pain online being unchanging over time/ still the	1	1

Name	Description	Files	References
	same as when pain started.		
I have a different circle of friends because of pain	Talking about having changed social groups/ circles entirely because of pain and associated disability.	1	1
I have been told that this particular pain will get worse	Reference to patella alta (knee joint condition) - advised by HCP that this pain will get worse over time.	1	1
I have had to cancel plans with my friends	References to making plans with friends, which then have to be cancelled at late notice due to pain.	3	5
I have met new friends online	Talking about meeting new friends via apps/websites/social media. References to meeting online friends via either pain-related and/or mental health-related issues.	3	4
I haven't been offered any pain medication	Statement that no pain medication has been offered by GP/ doctors. No medication tried.	1	2
'I just thought it was normal'	In vivo code. References to the initial belief that to experience chronic pain was normal/ a similar experience for everyone/ not abnormal.	3	9
'I just try to deal with it quietly'	In vivo code. References to not wanting other people to see that they have pain or pain-related issues. Includes using anonymous accounts or aliases.	3	4
I need stronger painkillers than what the GP can prescribe	Strong belief that UK GPs are powerless to prescribe stronger pain medication, and that they need to be taking some of the medications they have seen suggested by people online (US etc.) to manage their pain.	1	9
'I need to know that I'm not	In vivo code. Emphasis on searching online just	1	1

Name	Description	Files	References
the only one'	to know they are not the only young person with a pain condition; that they are not alone.		
I was given conflicting treatment advice	Reference to being given conflicting treatment advice, specifically about exercise (do less/ do more).	1	1
I was told it was 'stress-related', but that isn't the main trigger	Reference to someone else labelling pain as 'stress-related', and individual disagrees with this label/ believes stress is not the main trigger.	1	2
IBS-pain and period pain interacts	References to the interaction between IBS-pain and period pain e.g., period pain can worsen IBS pain and vice versa.	1	5
'If you've got more money you can handle illnesses better'	In vivo code. References to level of monetary income being important in enabling access to good treatments and services to help with chronic pain/illness.	2	5
I'm looking for advice that improves my QOL	Talking about looking for pain management advice with the aim to improve quality of life/ overall wellbeing (as opposed alleviating or reducing pain). Often juxtaposed with 'hoping for a cure', which is used in a humorous way.	4	6
Impact of COVID-19 on general wellbeing	Discussion of the impact of COVID-19 on general wellbeing, for example discussing the emotional impact of the lockdowns, lack of access to gyms/sports facilities, not being able to use public transport etc.	6	8
Implementing new techniques is challenging	References to trying a new pain management technique that they have been recommended by either a HCP or family/friend/partner, however, finding that implementation of the new technique is challenging.	5	17

Name	Description	Files	References
Instagram - health or gym inspo	Use of Instagram to find health and fitness or gym inspiration.	1	1
Instagram - information-seeking	Information-seeking using Instagram. Social media information-seeking often involves seeking experiential advice from others about symptoms and/or treatments for a painful condition.	7	20
Instagram - mental health inspo	Use of Instagram to find mental health inspiration and motivational/ encouraging/ positive mental health/ mental wellbeing posts.	2	3
Instagram - pain positivity	Use of Instagram to follow pages that post positive quotes and images about chronic pain.	1	2
Instagram - reading about others' experiences	Reading about other's personal experiences with pain via Instagram or reading conversations/ interactions between other users about their pain experiences. Emphasis on reading/ liking/ viewing rather than sharing one's own experience.	6	20
Instagram - sharing information and experiences	Sharing one's own personal experiences about chronic pain via Instagram, using either a personal account or an account built specifically for sharing experiences with chronic pain/ illness.	2	6
Instagram - the chronic pain 'community'	Finding a sense of community and support with chronic pain via Instagram, Emphasis on exchange of informational and/or empathetic support. Includes specific references to 'community' and references to making new friends via Instagram.	4	22
Instagram - workout and stretching videos	Reference to using/ saving workout and stretching videos that are circulated via	1	2

Name	Description	Files	References
	Instagram.		
Invisible conditions can be made visible through social media	References to wanting young people's stories of pain/ invisible illness to be shared via social media, followed by wider sharing to others who do not have a pain condition, to improve their understanding.	2	2
'It does affect my education but I still get my work done'	Overlaps with 'struggling to keep up with education/studying'. However, in this case, pain has not affected attainment/ achievement.	1	2
'It's just the curse of being a woman'	In vivo code. Others (doctors, parents) normalizing women's severe abdominal/menstrual pain as to be expected. Refusal/ apprehension to investigate/ treat the pain.	2	4
'I've lost a lot of my friends'	Talking about the loss of certain friendships or friendship groups due to the impact of pain.	3	3
'Keep the medication perfectly consistent'	In vivo code. Reference to keeping arthritis medication consistent for it to be effective.	1	1
Lack of education and understanding of invisible illnesses	Talking about a general lack of education and public understanding of invisible illnesses, such as pain conditions. Emphasis on that there needs to be more education and resources available for others who do not understand.	3	10
Lack of pain psychology services	References to a lack of availability of, or a lack of referral to, specific services for pain psychology. These individuals express that generic talking therapies are not appropriate because emotional issues would not be there if it weren't for chronic pain.	4	9
Lack of social interaction	Talking about a lack of social interaction due to	4	5

Name	Description	Files	References
during COVID-19	COVID-19 restrictions.		
Learning from personal experience	References to learning how to manage pain/ ways to reduce pain specifically from personal experience of what has worked and what has not.	3	6
Little to no impact on studying	Reference to pain having little to no impact on studying/ education.	1	1
Looking for realistic content that is also uplifting	References to looking for social media content that strikes a balance between being realistic about the impact of pain and being uplifting/ motivational.	2	3
Lots of different doctors	References to having seen lots of different doctors or specialists over the course of several months/ years.	6	13
Making adjustments	Making physical adjustments or using support equipment e.g., braces/ cane/ migraine glasses, to help increase comfort and reduce pain.	11	25
Making pain resources more aesthetically pleasing	Statement that it would be nice if pain resources were more aesthetically pleasing and print-friendly, Comparison made to mental health resources being more visually appealing.	1	2
Medical professionals don't believe in my condition	Reference to not feeling believed by medical professionals, and that there is still stigma in medicine regarding chronic pain conditions.	1	1
Medication description	Describing a medication name, label, or purpose. This includes specific pain medications, antidepressants, and any other medication taken in relation to managing the pain condition.	13	20

Name	Description	Files	References
Medication helps with engaging in physical activity	Reference to medication being helpful to complete physical activity goals. Specific reference to Duke of Edinburgh Award.	1	1
Medications reduce pain	Statements that medications do work to reduce level of pain severity (though usually do not alleviate pain entirely).	11	21
Mental health comorbidities	Talking about mental health comorbidities that have been treated independently/ diagnosed independently by a healthcare professional. Includes anxiety, depression, stress disorders, OCD, panic attacks, and many more.	8	20
Mental health support online	References to websites (sometimes in combination with apps/ social media) that are used specifically for mental health support e.g., Mind. Specific mention of mental health apps is also coded under mental health apps - other.	4	11
Mindfulness - very difficult when pain is high	Reference to mindfulness being difficult to engage in when pain intensity is high.	1	1
Mindfulness hasn't helped me	References to have tried mindfulness and not liked it, found it a struggle to 'be mindful'. Found that mindfulness did not help them/ would not try it again.	6	9
More free mindfulness and relaxation resources	Expression of need for more free/ cheaper meditation and mindfulness resources.	1	2
More information for young people specifically	Expressions of a need for information tailoring (online) towards young people. Several references to the NHS website needing to address this issue.	5	9
More online information and support for patella alta	More online information and support is needed specifically surrounding the condition patella alta	1	2

Name	Description	Files	References
	(a knee joint condition that causes pain).		
'My own search history has changed'	In vivo code. References to internet search history changing as pain changes, or as more information is gained about the pain condition and how to manage it.	6	7
My parent(s) panicked about potential diagnoses	Parental panic about a potential diagnosis of chronic illness (reference to chronic fatigue), having known someone else who has a diagnosis.	1	1
My school was not supportive	Reference to the school being non-supportive and seeing young person's pain as an 'excuse' not to attend.	1	1
My teacher was supportive	References to an individual teacher being supportive and helpful with pain issues. Provision of tangible and empathetic support.	2	3
My university supports me	References to the young person's university providing support with pain including access to support services, and extra time in exams.	5	5
My workplace are understanding	Reference to the workplace being understanding of physical pain condition and making adaptions for employee.	1	1
NHS waiting times	Discussion of NHS waiting times with the emphasis that there are long waiting times for appointments with primary or secondary care services. Discussion of waiting times for psychological therapies is coded separately under 'accessing psychological therapies'.	4	6
NHS website - COVID-19 banners are off-putting	Reference to COVID-19 banners (yellow headers) on the NHS website being off-putting when searching surrounding different condition.	1	1

Name	Description	Files	References
NHS website - directing friends to read information	Reference to directing friends to read information on the NHS website, which is easy for them to do.	1	1
NHS website - information-seeking	Information-seeking on the NHS website. This could be information-seeking about the cause, symptoms, or treatment for a painful condition, which are often sought in parallel.	21	55
NHS website - accessible, easy to use	References to the NHS website being easy to use/ navigate, accessible for everyone, clear to read, and easy to understand.	8	10
NHS website - basic or vague	References to the NHS website, being basic, vague, too simplistic, or not providing enough information about the specified condition.	13	20
No support with the emotional impact of a pain diagnosis	Discussion about the lack of support with the emotional impact of a chronic pain diagnosis. A lack of any signposting, information or reassurance given from the diagnosing doctor. No help with accepting pain chronicity.	1	4
No symptoms at the Drs appt.	Reference to the irony of not showing any symptoms at the time of the medical consultation/ doctor's appointment.	1	3
No 'unhelpful' resources	No unhelpful resources if you use common sense/ avoid irrelevant websites	9	10
Normalised GP visits due to a different condition	GP visits are normalised due to attending regularly for another separate condition (heart condition).	1	1
Online GP	Using/ describing use of an online, private GP.	1	2
Online health information is readily accessible	Talking about online health information being readily accessible in instances where a face-to-	2	2

Name	Description	Files	References
	face GP appoint is not available or is not immediately necessary.		
Online healthcare should be freely available to everyone	Discussion that good online healthcare should be freely available to everyone, or at least discounted. References to online GPs and advanced activity tracking such as Fitbit.	1	3
Online meditation classes	Taking online meditation classes that have been adapted from in-person due to COVID-19.	1	1
Online physiotherapy	Engaging with online physiotherapy, usually adapted from face-to-face physiotherapy due to COVID-19.	3	5
Online predators taking advantage of pain diagnoses	Specific references to predatory behaviour online via social media such as offering to give a massage to help pain etc.	1	2
Online psychological therapy	Engaging in online psychological therapies (talking therapy).	1	2
Other health websites - information-seeking	Information-seeking using other mentioned health websites. This could be information-seeking about the cause, symptoms, or treatment for a painful condition, which are often sought in parallel.	6	11
Other people normalise my pain	References to other people normalising the young persons' pain e.g., 'everybody experiences this' or 'I have that as well, it's normal'.	4	5
Other symptoms	Additional symptoms that relate to the pain condition - descriptions. Much of the content coded relates to Ehlers-Danlos Syndrome (a connective tissue disorder) and hypermobility, as well as the additional symptoms of	13	36

Name	Description	Files	References
	fibromyalgia, secondary headaches etc.		
Pain and fatigue	Talking about the relationship between pain or the pain condition and levels of fatigue/ energy.	11	19
Pain and sleep	References to having trouble getting to sleep or staying asleep because of physical pain. Some individuals in the fibromyalgia group talk about how they have been given medication to improve sleep, which has helped with overall pain and fatigue levels.	9	17
Pain changing over time	Long-term changes over the course of the persons' pain condition/ pain experience. The difference between pain then and pain now.	18	32
Pain is still the same	Stating that pain has stayed the same over time.	2	2
Pain sensation - allodynia	Reference to experiencing allodynia (hypersensitive pain sensation that occurs when touching the skin).	1	1
Pain-related worry	References to specific pain-related worries, which are described as worries or thoughts, usually 'what will happen if/when'.	8	9
Parents go to the GP with me	Statement that parents go to the GP with young person.	1	2
Parents have a 'traditional' view of pain management	Reference to parents having a traditional view of pain management and discouraging meditation/ mindfulness/ psychological strategies.	1	1
Parents relay online health information to me	Statement that parents look online and relay health information to the young person, rather than the young person directly accessing resources themselves.	1	3

Name	Description	Files	References
People around me don't understand	Perceived lack of understanding that people in the young persons' life do not understand their pain and the impact it has on their life. This could be friends, family members, colleagues etc. Links with code 'select family and friends are supportive.'	10	26
People can be nasty online	References to coming across people in online forums/ groups who are generally nasty towards others or make hurtful comments/ insults.	2	3
People spread misinformation online	Reference to people spreading misinformation online. Specific reference to anti-vaxxers.	1	2
PF - impact on physical activity	References to the pain conditions' impact on overall physical activity. For example, reducing physical activity due to pain or pain being a problem during regular physical activity, such as walking.	18	49
PF - routine tasks and self-care	References to the pain conditions' impact on daily tasks and self-care. For example, inability to shower, or put jeans on, or carry a bag. Struggling to write/ type is coded separately.	14	24
Physio - helpful but not tailored enough	Discussion that although aspects of physiotherapy can be helpful, the individual believes their physiotherapy programme is not tailored enough for their specific needs.	2	3
Physio - very difficult when pain is high	Discussion that engaging with physiotherapy is very difficult when pain severity is high/ pain flare present.	2	5
Physio 'a godsend'	References to physiotherapy being a really important, crucial aspect of the individuals' pain management plan. 'a godsend' coded in vivo.	3	7

Name	Description	Files	References
Physio 'I don't gel with that'	References to physiotherapy exercises/ advice that has been given, and finding that it is not helping with pain, or making a choice to not engage with it for specific individual reasons.	4	9
Pinterest - information-seeking	Using Pinterest to information-seek, as well as save, pain management resources.	1	2
Pinterest - relatable quotes	Use of Pinterest to look at 'relatable' pain-related quotes.	1	1
Pop culture pain inspirations	Talk about celebrities or public figures journeys as told online/ through social media. Specifically, Lady Gaga mentioned a few times in relation to Fibromyalgia.	2	7
Popping, cracking and subluxations	References to joint popping, cracking, clicking and subluxations (often referred to as dislocations). Usually present with hypermobility/ EDS.	5	7
Presenting to A&E with pain	Experiences of presenting with pain at A&E.	3	5
Prioritising other problems over pain	References to prioritising treatment/ management of other health or mental health problems over chronic pain.	2	5
Prioritising pain over other problems	Prioritising physical pain problem over addressing issues with mental health and/ or social life.	2	2
Psychological therapy	References to receiving psychological therapy/ talking therapies/ CBT.	8	15
Psychological therapy - CAMHS	Reference to receiving psychological therapy under CAMHS for pain-related issue.	2	5

Name	Description	Files	References
Reddit - an appropriate platform for younger people	Reference to Reddit being a more age-relevant platform for younger people compared to other health forums.	1	1
Reddit - information-seeking	Information-seeking using Reddit, which is similar to online forums or message boards, however, is also considered as social media. Information-seeking on Reddit often involves seeking experiential advice from others about symptoms and/or treatments for a painful condition.	2	5
Reddit - reading about others' experiences	Reading about other's personal experiences with pain via Reddit (forum-based social media) or reading conversations/ interactions between other users about their pain experiences. Emphasis on reading/ liking/ viewing rather than sharing one's own experience.	1	4
Remote working, studying and pain COVID-19	Talking about exacerbation of pain-related difficulties as a result of working/studying from home during COVID-19.	2	4
Resorting to private healthcare	Resorting to private healthcare for reasons including NHS waiting times, access to more tailored services than what the NHS can provide, or not meeting criteria for a certain service.	7	15
Rest is important	References to rest being important in terms of taking breaks and ensuring to allocate recovery time in relation to specific activities and/or generally balancing rest and activity.	8	16
Risking pain to achieve your goals	Talking about risking a backlash of pain to achieve a specific personal goal. Specific reference to Duke of Edinburgh Award.	1	1

Name	Description	Files	References
Risking pain to do things you enjoy	Talking about risking a backlash of pain to take part in activities that bring enjoyment or to continue to take part in activities that the individual is passionate about.	3	8
Running out of options	References to running out of treatment options for pain management. Includes references to treatments not working fully and not being offered anything further, and statements that there are limited options available.	10	13
Saving useful resources for later	References to saving/pinning/storing pain management resources found online, so that they can be returned to later/ when needed.	4	4
Searching for a diagnosis	References to searching for a diagnosis either via medical services or online. Several descriptions of wanting answers, pushing for a diagnosis from HCPs, and emphasising that they want to know what the problem is so they can get the right treatment.	15	31
Seeing the school counsellor	References to seeing the school counsellor to talk about pain and related issues. Particularly issues they experience at school.	2	3
Select family and friends are supportive	References to select friends and/or family members being supportive around the pain condition. Often this is a partner or 1 or 2 members of a family. Sometimes discussed in contrast with other friends and family who normalise pain or are dismissive.	13	22
Self-blaming	Expressions of self-blame for pain experience. Thoughts that they might have done something to deserve to have pain, or that they were just being lazy in some way.	3	6

Name	Description	Files	References
Self-inflicting pain in an attempt to desensitize	Attempting to desensitize the pain sensation via self-harming strategies to create temporarily increased pain sensation in the painful limb/ area.	1	3
Self-management - non-pharma physical	Use of heat and cool packs, massage, TENS for self-managing pain.	18	39
Self-management - relaxation, meditation, mindfulness	Use of relaxation, meditation, or mindfulness to self-manage pain or issues related to pain, such as sleep and stress. Mindfulness apps are layered as a separate code.	20	42
Self-management - stretching, strengthening and yoga	References to self-managing pain by stretching, completing strengthening exercises, and several references to yoga (which can also be for relaxation and a combination of stretching/ strengthening).	11	20
Self-management sometimes helps, sometimes doesn't	Statement that self-management strategies are being used, but that they only help sometimes.	1	1
Social media is a 'highlight reel'	References to social media being a 'highlight reel' or not a portrayal of 'real life'	2	3
Social media is readily accessible	References to social media and the internet being readily accessible, and that they can always turn to the internet for support and advice for pain, or mental health, as needed.	3	4
'Some people get very competitively ill'	Discussion of some individuals on social media turning pain severity and impact into a competition, particularly comparing their experience to others i.e., 'whose got it worse'.	4	10
Struggling to keep up with studying + attendance	References to struggles keeping up with school/college/university workloads. Includes	10	24

Name	Description	Files	References
	references to reduced attendance		
Struggling with work	Talking about pain-related issues with engaging with work/ employment. Several references to needing more breaks than colleagues, as well as not being able to take on a full-time role.	7	15
Struggling with writing + typing	References to struggling with completing tasks that involve writing or typing, due to hand and wrist pain.	6	10
Surgery can potentially make things worse	HCP advice given that surgery (knee surgery - patella alta) can potentially create more problems and make pain worse.	1	1
Taking nutritional advice from HCPs on board	Implementing nutritional advice given by healthcare professionals in relation to IBS and functional abdominal pain disorders.	2	4
Taking nutritional advice from non-HCPs	References to taking nutritional advice from non-healthcare professionals online.	1	2
Taking online information to the GP	References to taking information from either the NHS website, or other alternative health websites, with them to the GP appointment. This may be physically printed out/ electronic document or may be that they have a list of specific symptoms they are planning to mention.	7	11
Targeted ads for pain relief products	Reference to online advertisements for pain relief products, usually with a link to the website where you can purchase the product. These adverts are often targeted based on internet search history.	1	6
The changing digital world	Talking about how the digital world has rapidly changed, and that there is much more information and more support resources that	7	13

Name	Description	Files	References
	can be accessed online now compared to several years ago. Referenced frequently by those who have had pain for many years.		
The relationship between mood and pain	Talking about the relationship between mood and pain, and how the two are inter-related. Some individuals discuss that they do not fully understand the relationship / see how the two are related, however, most emphasise the importance of this relationship.	13	29
There is no treatment plan	References to there being no treatment plan, only management, with the emphasis that self-management is necessary because of this.	1	2
Tracking and recording pain	References to tracking or recording pain severity and pain-related symptoms, with the idea that it either is or could be helpful for pain management.	6	12
Transitioning from paediatric to adult healthcare	Discussion of 16-18 years transitional stage from paediatric to adult healthcare.	1	2
Traumatic experiences	Talking about pain-related traumatic experiences.	2	6
Treatment misinformation - dangerous to impressionable people	Stating that online misinformation regarding remedies and treatments for pain can be very dangerous to impressionable/ less educated people who might cause further problems for themselves by taking incorrect advice.	1	2
Treatments help, but only temporarily	References to pain management being frustrating because treatments only help temporarily, and the same strategies need to be repeated over and over.	3	3

Name	Description	Files	References
Trial and error treatment	Reference to treatment for the painful condition feeling as if it is 'trial and error' rather than a treatment plan.	1	4
Trustworthy information sources	References to a website or internet-based resource being trustworthy or reliable for health information either as a standalone or compared to other websites.	17	38
Trying to understand triggers	References to attempting to understand and avoid triggers that onset a pain flare. Frequently referenced by those experiencing chronic migraines or headaches. Triggers talked about mainly include light/ screens, and food and drinks.	7	14
Trying treatments suggested online	Trying out treatment advice/ management strategies from non-HCPs suggested online. Usually, suggestions found on forums and blogs.	7	8
Tumblr - light-hearted chronic pain humour	Reference to using Tumblr to follow light-hearted chronic pain humour. This is different to 'realistic content that is also uplifting' in the sense that this is only sought for the comedic value.	1	2
Turning to my partner first	Turning to a partner as the first point of support when experiencing high pain levels or generally turning to them before anything/ anyone else (parents, internet).	1	1
Turning to parents first	Turning to parents as a first line of support with pain, before other support sources (internet, partner).	5	6
Turning to the internet first	Turning to the internet as a first line of support with pain and pain-related issues. This is done	11	11

Name	Description	Files	References
	before turning to anyone/ anywhere else (parents, partner, GP). Relates to code 'taking online information to the GP'.		
Twitter - information-seeking	Information-seeking using Twitter, which is considered as social media. This could be information-seeking about the cause, symptoms, or treatment for a painful condition, from an experiential and/or medical/scientific perspective.	1	2
Twitter - reading about others' experiences	Reading about other's personal experiences with pain via Twitter or reading conversations/ interactions between other users about their pain experiences. Emphasis on reading/ liking/ viewing rather than sharing one's own experience.	1	2
Understanding medication by searching online	Talking about attempts to better understand pain medication the individual has been prescribed by searching online.	3	7
Understanding pre-disposing factors by searching online	Understanding pre-disposing factors e.g., genetic factors by searching online. Reference is specifically to lactose intolerance.	1	1
Understanding that pain disorders are interlinked	Statement of understanding that having one pain disorder makes your vulnerable to others.	1	1
Unpredictable pain	Describing pain as different on different days, ups and downs of pain on the same day, bad pain days verses good pain days	13	25
Use of disability services	References to using/accessing disability services to help with pain-related issues.	5	10
We need medical professional advocacy	Reference to needing medical professionals to show their support and advocacy for chronic	1	1

Name	Description	Files	References
online	pain conditions online.		
WebMD - information-seeking	Information-seeking on WebMD. This could be information-seeking about the cause, symptoms, or treatment for a painful condition, which are often sought in parallel. WebMD has a symptom checker.	10	20
WebMD - unhelpful	References to WebMD being unhelpful by being either dismissive of the impact of hypermobility syndromes, or the symptom checker being anxiety-provoking.	3	8
Websites downplayed my pain condition	References to websites downplaying or dismissing the impact of Ehlers Danlos/ hypermobility syndromes.	2	3
'what works for them might not work for me'	In vivo code. Recognition that treatments that work for one person may not work for another.	2	4
wikiHow - information-seeking	Information-seeking using wikiHow. This is usually in the form of 'how to treat' a specific ailment, using a step-by-step guide, with images.	1	1
Women understand women's pain	Reference to women understanding pain related to gynaecological issues better than a male doctor could.	1	2
Worries about medication dependence	Expression of worry about taking medication frequently/ becoming dependent.	3	4
You can't replace seeing a doctor in-person	Relates to accessing healthcare during COVID-19 code. Even though the NHS website is good, and telephone appointments are available, individual expresses that you can't replace a doctor getting a holistic view in-person, and that in-person interaction is also important to feel	1	1

Name	Description	Files	References
	understood as a patient.		
'You have to learn to balance all your activities'	References to the importance of learning balance or pace all your activities, including physical activity, however also including establishing a general balance of e.g., work-life.	5	15
You need to be organised to manage pain	References to personal organisation being important to manage pain effectively. Includes keeping medication on-hand and creating reminders to do exercises such as physio or yoga etc.	6	8
'Young people shouldn't have pain'	References embodying the view from others' that 'young people shouldn't have pain'. Includes references to doctors thinking young people are 'exaggerating', as well as friends/ family members stating the young person is 'too young' for said pain condition. Sometimes this view is internalised.	11	18
Young people, weight-related issues and pain	Talking about how the population weight-related issues might reflect in more young people starting to have problems with joint pain (due to obesity/ being overweight).	1	2
YouTube - easy to search	Statement that YouTube is easier to search comparably to Instagram.	1	1
YouTube - information-seeking	Information-seeking using YouTube, which is considered as social media. This could be information-seeking about the cause, symptoms, or treatment for a painful condition, from an experiential and/or medical/scientific perspective.	10	16
YouTube - listening to others' experiences	Listening to other's personal experiences with pain via YouTube videos. This can overlap with	8	13

Name	Description	Files	References
	information-seeking on YouTube e.g., what was someone's experience of getting a diagnosis and how did they get it?		
YouTube - parent relaying advice from videos	Reference to a parent looking for informational advice on YouTube and relaying the information they find to the young person.	1	2
YouTube - relaxing soundtracks and meditation	Use of YouTube to source relaxing soundtracks and/or meditations, in order to facilitate self-management of pain or pain-related issues.	4	7
YouTube - yoga + stretching	Use of YouTube to source yoga and stretching videos, in order to facilitate self-management of pain.	5	7
